# Virus-induced perturbations in the mouse microbiome are impacted by microbial experience

**DOI:** 10.1128/msphere.00563-24

**Published:** 2025-02-13

**Authors:** Shanley N. Roach, Wendy Phillips, Lauren M. Pross, Autumn E. Sanders, Mark J. Pierson, Ryan C. Hunter, Ryan A. Langlois

**Affiliations:** 1 Department of Microbiology and Immunology, University of Minnesota, Minneapolis, Minnesota, USA; 2 Minnesota Supercomputing Institute, University of Minnesota, Minneapolis, Minnesota, USA; 3 Department of Microbiology and Immunology, University at Buffalo, Getzville, New York, USA; Nanjing University of Chinese Medicine, Nanjing, Jiangsu, China

**Keywords:** microbiome, influenza virus, dysbiosis, dirty mice

## Abstract

The bacterial microbiome has a major impact on health and can shape metabolism, host tolerance, immune responses, and the outcome of future infections. The bacterial microbiome is highly variable between individuals. Specific pathogen-free animals have reduced microbiome diversity, making it difficult to evaluate the impact of infection-induced microbiome disruption that would be observed in free-living animals, including people. Mice are commonly used as a preclinical model but unfortunately often fail to predict translation success or failure, particularly for immune and infectious disease-targeting therapies. Here, we utilize pet store mouse cohoused “dirty” mice with diverse microbial experience to explore how host variability and infection may be interacting to drive unique microbiome changes. We found that cohoused animals had significantly increased bacterial diversity in the small intestine and cecum but not in the large intestine. There were differentially abundant taxa between clean and dirty animals in all three tissues. After infection with influenza A virus, samples clustered by both housing condition and infection status in the cecum and large intestine, while small intestine samples clustered predominantly by infection. Altogether, these results highlight the differential impact of housing, infection, and interaction between the two in dictating community composition across the gastrointestinal microbiome.

**IMPORTANCE:**

Traditionally housed pathogen-free mouse models do not fully capture the natural variability observed among human microbiomes, which may underlie their poor translationally predictive value. Understanding the difference between pathogen-induced shifts in the bacterial microbiome and natural microbiome variance is a major hurdle to determining bacterial biomarkers of disease. It is also critical to understand how diverse baseline microbiomes may be differentially impacted by infection and contribute to disease. Pet store cohoused “dirty” mice have diverse microbial experiences and microbiomes, allowing us to evaluate how baseline variation, infection, and interaction between the two impact the microbiome.

## OBSERVATION

Bacterial microbiota colonize mucosal sites throughout the body of animals and can significantly impact health (1). Specific pathogen-free (SPF) mice dominate preclinical research studies. Due to their sterile housing conditions, they have a dramatically altered intestinal microbiome compared to free-living mice (2–5), which can impact subsequent infections and immune responses (6, 7). It is becoming increasingly clear that viruses and bacterial microbiota can influence one another. For example, experimental respiratory viral infections in mice can impact the microbiome at a distal site, like the gastrointestinal tract (8–11). This phenomenon has also been observed in humans (12, 13). Bacterial microbiomes in humans can vary significantly based on geography, diet, and health conditions and may play a role in shaping virus-induced perturbations (14, 15). This natural variability is difficult to capture in traditionally housed SPF mice. This presents a major hurdle not only to identifying conserved virus-induced perturbations but also to determining how host microbiome variability and infection may be interacting to drive unique microflora changes. We hypothesized that virus-induced perturbations in the microbiome would be different between SPF and pet store cohoused (CoH) “dirty” mice. Here, we compared the intestinal microbiomes of SPF and dirty mice at steady state and during acute influenza A virus (IAV) infection. We found that cohoused mice had a significant increase in alpha diversity and differences in taxonomic abundances between infected and uninfected mice across anatomical sites. IAV infection resulted in an interaction effect between housing condition and infection in driving microbiome alterations. Altogether, these results highlight the utility of the pet store CoH model for studying virus-induced microbiome perturbations.

### Pet store cohoused animals have increased bacterial microboiome diversity in the gastrointestinal tract

**Fig 1:**
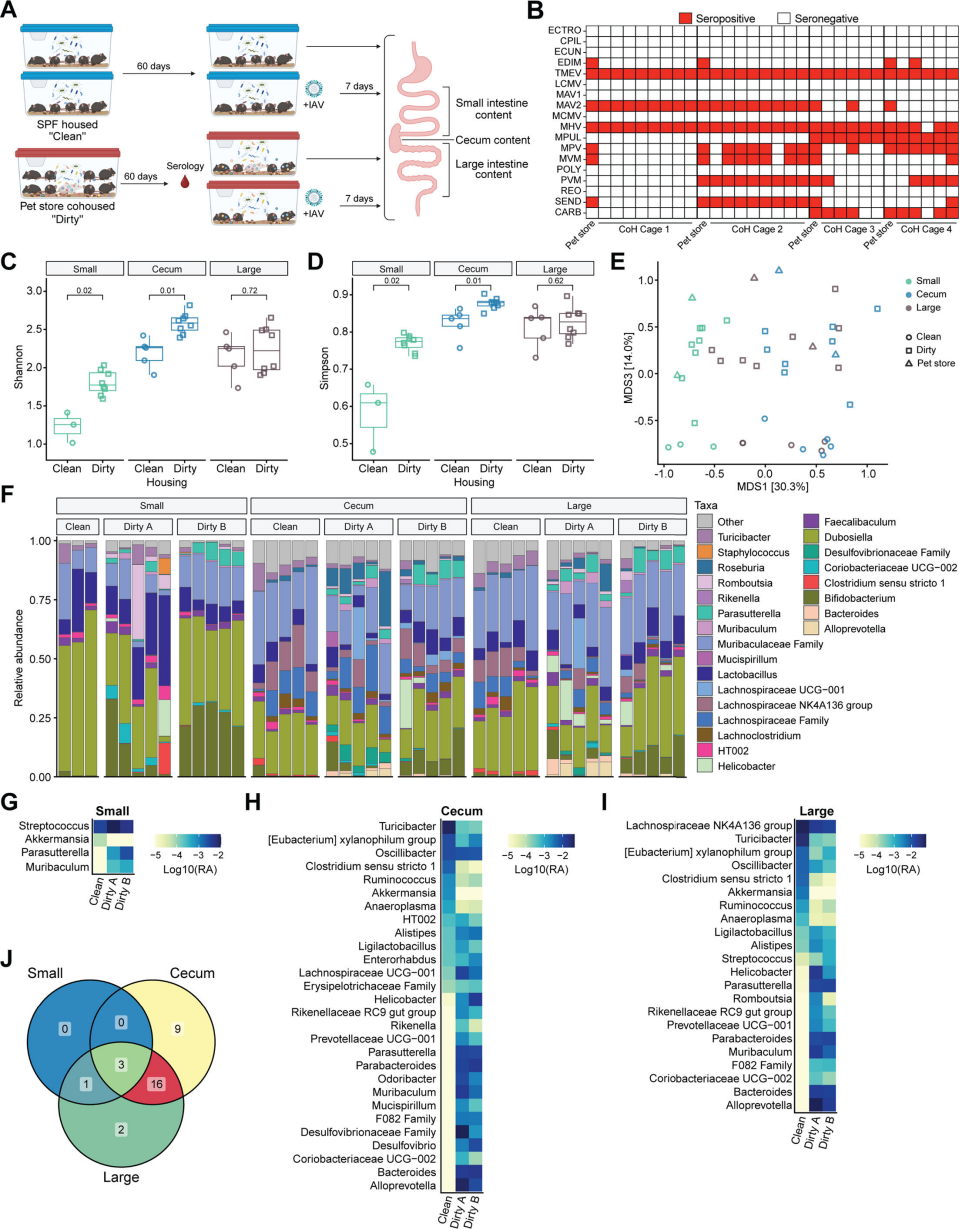
(**A**) Schematic of experimental setup and samples collected for 16S rRNA gene sequencing. CoH cages were set up with *n* = 1 pet store and *n* = 8 B6 animals per cage. See the text for a more detailed experimental design. (**B**) Serology of all pet store and CoH animals after 60 days post-CoH. See the text for pathogen acronym definitions. (**C and D**) Shannon’s (**C**) and Simpson’s (**D**) alpha-diversity indices of bacterial diversity across microbiome tissue sites in uninfected SPF (clean) and CoH (dirty) animals. Adjusted *P*-values determined by Wilcoxon rank sum test between clean and dirty mice for each tissue and displayed within the plot. (**E**) Principal coordinate analysis (PCoA) of Bray–Curtis dissimilarities of bacterial microbiome (lowest taxa classification) across all tissues in uninfected clean, pet store, and dirty mice. Shape denotes housing identity and color indicates tissue sampled. (**F**) Stacked bar plot of relative bacterial abundance (lowest taxa classification) of uninfected samples, pet store animals are the farthest left bar in each group. Dirty samples were separated by cage setup noted in panel B: cage A is from CoH cage 1 and cage B is from CoH cage 2. (**G–I**) Differentially abundant taxa (adj. *P* < 0.05) between housing conditions were identified using MaAsLin2 and the average log10 relative abundance (RA) plotted by cage for small intestine (G, “Small”), cecum (H, “Cecum”), and large intestine (I, “Large”). (**J**) Venn diagram showing the number of differentially abundant taxa (adj. *P* < 0.05) shared between tissues. (C–J) Data generated from uninfected mice in experiment 1 from CoH cages 1 and 2.

In total, we performed two independent pet store CoH experiments, each with age-matched clean controls (Fig. 1A). Serology screening after 60 days of CoH revealed exposure to several viral and bacterial pathogens (Fig. 1B). Pathogen exposure varied both between cages set up simultaneously and between two independent experiments. This bolsters what previous studies have reported and highlights the potential for pet store mice to provide a rich microbial experience to B6 mice (7, 16–18). To evaluate microbiome changes during housing, the small intestine, cecum, and large intestine content from half the CoH dirty mice in each cage from experiment 1 and age-matched SPF clean mice were evaluated by 16S rRNA gene sequencing.

Shannon and Simpson alpha diversity indices were significantly higher in the small intestine and cecum of dirty mice but not the large intestine (Fig. 1C and D). We used Bray–Curtis dissimilarities to compare bacterial community composition and plotted samples using principal coordinate analysis (PCoA). Sample clustering was driven by both tissue and housing status (Fig. 1E). Community composition significantly differed between clean and dirty mice for the cecum (*F* = 3.6038, *P* = 0.0072) and large intestine (*F* = 3.4770, *P* = 0.0113) but not the small intestine (*F* = 2.0175, *P* = 0.1234). This could be due to the lower sample size and therefore less statistical power. There were taxonomic differences across housing and tissues (Fig. 1F). Microbiome multivariable association with linear models 2 (MaAsLin2) testing revealed differentially abundant taxa between clean and dirty samples in all three tissues, with the greatest number seen in the cecum and large intestine (Fig. 1G through I). Many of these differentially abundant taxa were more prevalent in dirty versus clean samples (Fig. 1G through I; Fig. S1A through C) and were shared across tissues (Fig. 1J). These results demonstrate that gastrointestinal microbiome diversity and composition are altered by CoH.

### Infection-driven microbiome alterations are differentially influenced by pet store mouse cohousing based on tissue location

**Fig 2:**
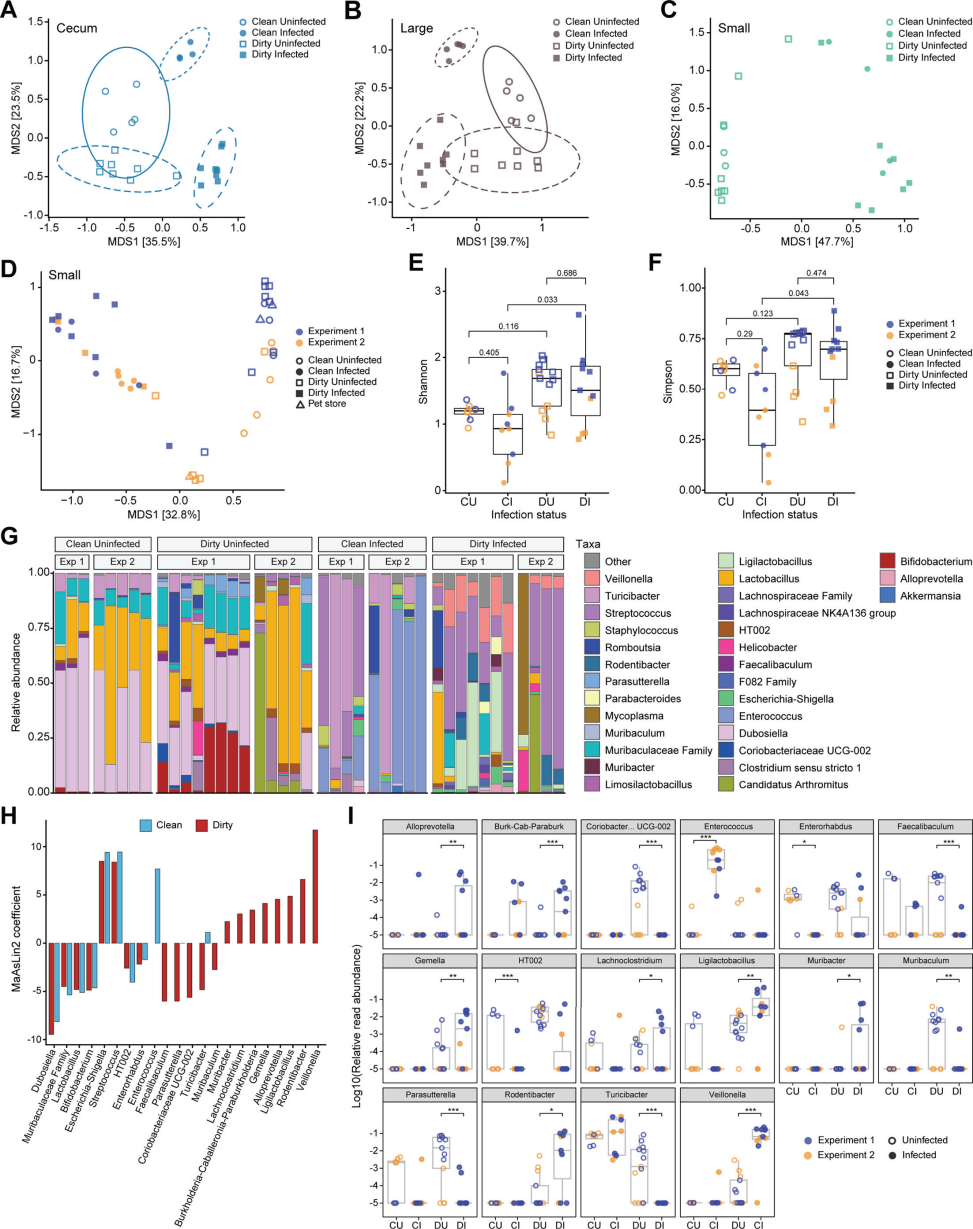
(**A–C**) Principal coordinate analysis (PCoA) of Bray–Curtis dissimilarities calculated for uninfected and infected ceca (A, “Cecum”), large intestine (B, “Large”), and small intestine (C, “Small”) of animals from each housing condition, excluding pet store mice. Ellipses were calculated and included for the cecum and large intestine but not the small intestine due to lower sample number of infected clean animals. Shape denotes housing identity, and fill indicates infection status. (**D**) PCoA axes 1 and 2 of Bray–Curtis dissimilarities for combined small intestine samples. Shape depicts housing condition, color indicates experiment/cage of origin, and fill shows infection status. See Fig. S1 for axes 1 and 3. (**E and F**) Shannon’s (**E**) and Simpson’s (**F**) alpha-diversity indices of combined small intestine separated by housing condition and infection status, excluding pet store mice. Shape depicts housing condition, color indicates experiment, and fill shows infection status. Wilcoxon rank sum test adjusted *P*-values are denoted for relevant pairwise combinations. (**G**) Stacked bar plot of combined small intestine relative bacterial abundance (lowest taxa classification) of uninfected and infected clean and dirty samples, excluding pet store mice. (**H**) MaAsLin2 effect size of differentially abundant taxa (adj. *P* < 0.05) between uninfected and infected samples for each housing condition. A bar for both housing conditions indicates that at least one, but not necessarily both, had an adj. *P* < 0.05. (**I**) Log10 relative abundance of taxa that were differentially abundant by infection status in only one of the two housing conditions. Color depicts experiment, and fill indicates infection status. Adj. *P* > 0.05 = ns, adj. *P* < 0.05 = *, adj. *P* < 0.01 = **, adj. *P* < 0.001 = ***. “CU” =clean uninfected, “CI” =clean infected, “DU” =dirty uninfected, “DI” =dirty infected. (A–C) Data generated from uninfected and infected mice in experiments 1 from CoH cages 1 and 2, which were set up at the same time. (D–I) Data generated from uninfected and infected mice in experiments 1 and 2 from CoH cages 1–4.

To evaluate if microbiomes would differ between clean and dirty infected mice, we infected a cohort of animals from experiment 1 with IAV and collected the small intestine, cecum, and large intestine content for 16S rRNA gene sequencing at 7 days post-infection. Bray–Curtis dissimilarity was plotted by PCoA. Cecum and large intestine samples were separated both by housing condition and infection status (Fig. 2A and B), and there were many differentially abundant taxa between uninfected and infected samples in both housing conditions (Fig. S1D and E). Interestingly, small intestine samples were predominantly separated by infection status (Fig. 2C). Together, these data suggest a potential interaction effect between housing and infection on the cecum and large intestine microbiome composition, whereas the small intestine is potentially more altered by infection.

To further explore the impact of infection on clean versus dirty small intestine microbiomes, we combined the first experiment’s small intestine results with results from a second experiment of two pet store CoH cages and age-matched SPF animals. PCoA plots of Bray–Curtis dissimilarities showed strong sample separation by infection status along the first PCoA axis, which explained 32.8% of the variance in community composition (Fig. 2D). Community composition significantly differed between uninfected versus infected samples in clean (*F* = 14.5123, *P* = 0.001) and dirty (*F* = 10.8539, *P* = 0.001) conditions. Composition also differed between infected clean and dirty (*F* = 6.7116, *P* = 0.001) but not between uninfected clean and dirty (*F* = 2.5007, *P* = 0.064). With the combined data set, dirty uninfected samples no longer had significantly higher alpha-diversity than clean samples (Fig. 2E and F). Alpha-diversity did not differ by infection status in either clean or dirty samples, but the dirty infected diversity was significantly higher than the clean infected diversity (Fig. 2E and F). The beta-diversity results suggest that infection status contributed more strongly to community composition, while alpha-diversity was more correlated with housing status.

Based on inspection of relative abundances, the taxa most prevalent in uninfected versus infected animals differed for both clean and dirty samples (Fig. 2G). Interestingly, taxa prevalent in clean infected samples appeared to differ from those in dirty infected samples. To further explore this, we used MaAsLin2 to identify differentially abundant taxa between uninfected and infected samples for each housing condition and plotted the coefficient to visualize effect size. We found taxa differentially abundant during infection that were shared by both housing conditions and ones unique to each (Fig. 2H; Fig. S1G). Visualizing log10 relative abundance of taxa that were different by infection status in only one of the housing conditions further revealed housing-specific infection-induced microbiome changes (Fig. 2I). This suggests that, while infection is the dominant driver in community diversity and composition in clean and dirty mice, there still is some interaction between infection and housing contributing to infection-induced changes. Altogether, these results highlight the differential impact of housing, infection, and interaction of the two in dictating community composition across the gastrointestinal microbiome.

Previous studies exploring IAV infection-induced microbiome alterations conducted in SPF mice do not recapitulate the diversity seen in humans, making it difficult to characterize infection-specific changes and understand how baseline microbiome diversity impact changes during infection. The differences in pathogen exposure (Fig. 1B), immune activation (18), and baseline microbiome in pet store CoH animals provide a platform to explore the interaction between infection-driven microbiome shifts and diverse pre-infection microbiome compositions. Here, we demonstrate that dirty mice generally had a higher alpha-diversity and differentially abundant taxa compared with SPF clean animals (Fig. 1; Fig. S1A through C). We observed an interaction effect between housing condition and infection across the intestinal microbial community composition (Fig. 2A through D; Fig. S1D through F). These data suggest that microbiome variation across individuals at these tissues will lead to unique and varied shifts during IAV infection, with the small intestine microbiome highly susceptible to infection-induced alterations. Altogether, the diversity in the dirty CoH model can be leveraged to further study how varied microbiome and microbial exposures contribute to infection-induced microbiome alterations and explore factors that contribute to individual versus conserved infection-driven changes.

### Cohousing

Pet store mice were acquired from local pet stores and cohoused (CoH) with 8-week-old specific pathogen-free (SPF) C57BL/6J (B6) mice (The Jackson Laboratory) within a BSL-3 facility. One pet store mouse was CoH with eight B6 mice. Non-CoH age-matched B6 mice were maintained in the SPF facility until the time of infection, after which both uninfected and infected non-CoH cages were transferred to BSL-2 for the remainder of the experiment. The hard bedding material and rodent chow used in BSL-3 and SPF/BSL-2 were the same. Male B6 mice cannot be CoH with male pet store mice, as this creates significant animal welfare concerns due to fighting, aggression, and social defeat. Therefore, we could ethically only use female mice for CoH experiments. All experiments using mice were approved by the Institutional Animal Care and Use Committee (IACUC) at the University of Minnesota.

Experiment 1 had two dirty CoH cages (CoH cages 1 and 2) and two age-matched SPF cages. Both pet store mice were acquired at the same time. After 60 days, mice from each of the CoH cages were separated into two cages, and one of the two were infected. At the same time, one SPF cage was infected, and one cage was left uninfected. Experiment 2 had two cages each of dirty CoH (CoH cages 3 and 4) and age-matched SPF mice. Both pet store mice were acquired at the same time but were from a separate shipment of pet store mice than those in experiment 1. After 60 days, one cage from each housing group (CoH or SPF) was infected, and one remained uninfected.

### Serology

Mice were CoH for 60 days, then pet store and B6 mice were bled for immune phenotyping and serology screened using EZ-spot (IDEXX BioAnalytics) prior to proceeding with infection and microbiome analysis. The IDEXX serology panel screens for ectromelia virus (ECTRO), *Clostridium piliforme* (CPIL), *Encephalitozoon cuniculi* (ECUN), murine rotavirus (EDIM), Theiler’s murine encephalitis virus (TMEV), lymphocytic choriomeningitis virus (LCMV), murine adenoviruses (MAV1 and MAV2), murine cytomegalovirus (MCMV), murine hepatitis virus (MHV), *Mycoplasma pulmonis* (MPUL), murine parvoviruses (MPV), minute virus of mice (MVM), polyoma virus (POLY), pneumonia virus of mice (PVM), murine reovirus (REO), Sendai virus (SEND), and *Filobacterium rodentium* (CARB).

### IAV infections

After serology profiling, uninfected animals remained with the pet store mouse and mice that were to be infected were transferred to a new cage prior to infecting. BSL-3 CoH B6 and age-matched SPF mice were anesthetized using a weight-based dose of ketamine–xylazine delivered intraperitoneally (i.p.), then infected intranasally (i.n.) with a 40 PFU (sublethal) dose of influenza A/Puerto Rice/1934 (PR8). Uninfected and infected SPF mice were both transferred and housed in BSL-2 conditions for the remainder of the experiment.

### Sample collection

Mice were euthanized, and the entire gastrointestinal tract was removed from below the stomach and above the rectum. The small intestine, cecum, and large intestine were separated, and their contents were collected separately in sterile 1.5 mL Eppendorf tubes. Samples were flash frozen on dry ice and stored at −80°C until extraction.

### DNA isolation and sequencing

DNA was extracted from all sites using the Allprep Powerfecal DNA/RNA Kit (Qiagen). Small intestine, cecum, and large intestine contents were thawed on ice and mixed with buffer according to kit instructions and extracted. Buffer-only extraction controls were also processed. Extracted DNA from samples, buffer controls, and water-only submission controls were sent to the University of Illinois at Urbana-Champaign Roy J. Carver Biotechnology Center for 16S rRNA gene sequencing. The V4 region was amplified by PCR on a Fluidigm access array using primers V4 515F (5′-GTGCCAGCMGCCGCGGTAA-3′) and V4 806R (5′-GGACTACHVGGGTWTCTAAT-3′) and sequenced on the Illumina MiSeq V2 platform with 250 bp paired-end reads. Extraction buffer controls and water submission controls were also submitted for sequencing as controls but were below detectable threshold levels.

### 16S rRNA analysis

Amplicon sequence analysis was performed in R (v.4.3.2). Cutadapt (v.4.6) (19) was used to remove primer sequences and to remove reads not containing adapters. DADA2 (v.1.30) (20) was used to trim and filter sequences [truncLen = c(200,180), maxEE = c(2, 5)] and to correct sequencing errors by inferring a parametric error model. Reads were dereplicated, paired ends merged, and chimeric reads removed using default options. Sequencing yielded low coverage (<1,000 reads) from four samples in the first experiment, which were removed from analysis. Resulting amplicon sequence variants (ASVs) were assigned taxonomy using RDP Bayesian classifier (21) and the SILVA-138 taxonomy training set (release 138) (22). Sequences without classification at the phylum level or with fewer than 10 total read counts were discarded. All downstream analyses were performed with a taxa table with ASVs assigned to the lowest possible taxonomic level, which was either the genus or family level.

### Data visualization and statistical analyses

All data visualization and statistical analyses were performed with R (v4.3.2) software. For all analyses, taxa tables with ASVs were assigned to the lowest possible taxonomic level, which was either the genus or family level. Small intestine taxa tables were rarefied to 1,700 counts, and cecum and large intestine samples were rarefied to 30,502 counts prior to alpha- and beta-diversity analyses. Boxplots were generated using ggplot2 (v3.5.0). Heatmaps were plotted using pheatmap (v1.0.12). For stacked bar plots, any taxa with an abundance >5% was included in Fig. 1, and any taxa with an abundance >3% was included in Fig. 2. The phyloseq (v1.46.0) package (23) was used to calculate the Shannon and Simpson alpha-diversity indices. Differences between clean and dirty housing were tested with the Wilcoxon rank sum test. Dunn test was used as a wrapper to do Wilcoxon rank sum tests between pairwise combinations of the housing and infection groups. Adjusted *P*-values are shown in the plots. Beta-diversity analysis was performed with the package R packages vegan (v2.6-8) and microViz (v0.12.0) (24). Bray–Curtis dissimilarities were calculated, and samples were plotted using principal coordinates analysis (PCoA). PERMANOVA with 9,999 permutations was used to test for differences in community composition based on Bray–Curtis dissimilarities, with pet store samples removed, between housing conditions for each tissue separately. In Fig. 2, cecum and large intestine ordinations included ellipses with the ggplot2 function stat_ellipse. The small intestine clean group had too few samples for an ellipse to be calculated. The R program MaAsLin2 with default settings was used for differential abundance testing (25). Taxa that were not present at a relative abundance greater than 0.1% in at least two samples in that tissue type were removed prior to testing. Taxa that had an adjusted *P* < 0.05 were plotted for each tissue type. Log10 transformed relative abundances were used for plotting, with zero counts changed to a count of 1e−5 prior to log transformation for boxplots. For testing between housing conditions, only uninfected (excluding pet store mice) samples were used, and each tissue type was tested separately. For testing between infection status in cecum and large intestine samples, each initial cage for each tissue type was tested separately. For testing infection status in the combined small intestine data, default settings and fixed effects for cage were included in the model used to test for taxa with differential abundances between conditions, and taxa with an adj. *P* < 0.05 in either clean or dirty samples were plotted. The MaAslin2 coefficient, i.e., effect size, for each uninfected–infected comparison was also generated.

## Data Availability

Previously published software packages and versions used to analyze 16S rRNA sequence data are cited in the text. Sequence data were deposited and are available as FASTQ files in the NCBI sequence read archive under BioProject no. PRJNA1144438. Code used for analyses can be found at https://github.com/langloislab.

## SUPPLEMENTAL MATERIAL

Supplemental material msphere.00563-24-s0001.docx Figure S1.

ASM does not own the copyrights to Supplemental Material that may be linked to, or accessed through, an article. The authors have granted ASM a non-exclusive, world-wide license to publish the Supplemental Material files. Please contact the corresponding author directly for reuse.
