# Supplementary material for: Virus-induced perturbations in the mouse microbiome are impacted by microbial experience
Source: mSphere. 2025 Feb 13;10(3):e00563-24. doi: 10.1128/msphere.00563-24 (PMC11934326; doi:10.1128/msphere.00563-24)
Supplement: Supplemental material — Figure S1. [file msphere.00563-24-s0001.docx]

**Supplemental Figure 1. A.** Individual log10 relative abundance (RA) of differentially abundant taxa in clean versus dirty small intestine from Figure 1G. **B.** Individual log10 relative abundance (RA) of differentially abundant taxa in clean versus dirty cecum from Figure 1H. **C.** Individual log10 relative abundance (RA) of differentially abundant taxa in clean versus dirty large intestine from Figure 1I. **D.** Log10 relative abundance (RA) of differentially abundant taxa between uninfected and infected clean and dirty cecum with an adj. p-value < 0.05. **E.** Log10 relative abundance (RA) of differentially abundant taxa between uninfected and infected clean and dirty large intestine with an adj. p-value < 0.05. **F.** Principal Coordinate Analysis (PCoA) axis 1 and 3 from combined small intestine samples in Figure 2D. Shape depicts housing condition, color indicates experiment, and fill shows infection status. **G.** Log10 relative abundance (RA) of differentially abundant taxa between uninfected and infected clean and dirty combined small intestine samples with an adj. p-value < 0.05. A-C: data generated from uninfected mice in experiment 1 from CoH cages 1 and 2. D-E: data generated from uninfected and infected mice in experiment 1 from CoH cages 1 and 2. F-G: data generated from uninfected and infected mice in experiment 1 and 2 from CoH cages 1-4.
